# Species distribution and antimicrobial susceptibility of gram-negative aerobic bacteria in hospitalized cancer patients

**DOI:** 10.1186/1479-5876-7-14

**Published:** 2009-02-19

**Authors:** Hossam M Ashour, Amany El-Sharif

**Affiliations:** 1 Department of Microbiology and Immunology, Faculty of Pharmacy, Cairo University, Cairo, Egypt; 2 Department of Microbiology and Immunology, Faculty of Pharmacy, Al-Azhar University, Cairo, Egypt

## Abstract

**Background:**

Nosocomial infections pose significant threats to hospitalized patients, especially the immunocompromised ones, such as cancer patients.

**Methods:**

This study examined the microbial spectrum of gram-negative bacteria in various infection sites in patients with leukemia and solid tumors. The antimicrobial resistance patterns of the isolated bacteria were studied.

**Results:**

The most frequently isolated gram-negative bacteria were *Klebsiella pneumonia *(31.2%) followed by *Escherichia coli *(22.2%). We report the isolation and identification of a number of less-frequent gram negative bacteria (*Chromobacterium violacum*, *Burkholderia cepacia, Kluyvera ascorbata, Stenotrophomonas maltophilia, Yersinia pseudotuberculosis*, and *Salmonella arizona*). Most of the gram-negative isolates from Respiratory Tract Infections (RTI), Gastro-intestinal Tract Infections (GITI), Urinary Tract Infections (UTI), and Bloodstream Infections (BSI) were obtained from leukemic patients. All gram-negative isolates from Skin Infections (SI) were obtained from solid-tumor patients. In both leukemic and solid-tumor patients, gram-negative bacteria causing UTI were mainly *Escherichia coli *and *Klebsiella pneumoniae*, while gram-negative bacteria causing RTI were mainly *Klebsiella pneumoniae*. *Escherichia coli *was the main gram-negative pathogen causing BSI in solid-tumor patients and GITI in leukemic patients. Isolates of *Escherichia coli, Klebsiella, Enterobacter*, *Pseudomonas, and Acinetobacter *species were resistant to most antibiotics tested. There was significant imipenem -resistance in *Acinetobacter *(40.9%), *Pseudomonas *(40%), and *Enterobacter *(22.2%) species, and noticeable imipinem-resistance in *Klebsiell*a (13.9%) and *Escherichia coli *(8%).

**Conclusion:**

This is the first study to report the evolution of imipenem-resistant gram-negative strains in Egypt. Mortality rates were higher in cancer patients with nosocomial *Pseudomonas *infections than any other bacterial infections. Policies restricting antibiotic consumption should be implemented to avoid the evolution of newer generations of antibiotic resistant-pathogens.

## Background

Hospital-acquired (nosocomial) infections pose significant threats to hospitalized patients, especially the immunocompromised ones [1]. They also cost the hospital managements significant financial burdens [1,2]. Cancer patients are particularly prone to nosocomial infections. This can be due to the negative effect of chemotherapy and other treatment practices on their immune system [3]. Most of the previous studies with cancer patients have only focused on bloodstream infections. However, limited information is available regarding the spectrum and microbiology of these infections in sites other than the bloodstream, such as the urinary tract, respiratory tract, gastro-intestinal tract, and the skin. This is despite the fact that these infections are not rare.

Our group has previously studied the microbial spectrum and antibiotic resistance patterns of gram-positive bacteria in cancer patients [4]. In the present study, the microbial spectrum of gram-negative bacteria isolated from various infection sites in hospitalized cancer patients was examined. The spectrum studied was not limited to the most common gram-negative bacteria, but included less-frequent gram negative bacteria as well. Both patients with hematologic malignancies (leukemic patients) and patients with solid tumors were included in the study. Thus, the resistance profile of the isolated gram-negative bacteria was examined. In addition, we detected mortality rates attributed to nosocomial infections caused by gram-negative isolates.

## Materials and methods

### Patient specimens

Non-duplicate clinical specimens from urine, pus, blood, sputum, chest tube, Broncho-Alveolar Lavage (BAL), throat swabs, and skin infection (SI) swabs were collected from patients at the National Cancer Institute (NCI), Cairo, Egypt. The SI swabs were obtained from cellulitis, wound infections, and perirectal infections. For each specimen type, only non-duplicate isolates were taken into consideration (the first isolate per species per patient). Data collected on each patient consisted of demographic data including age, sex, admission date, hospitalization duration, ward, and sites of positive culture. Selection criteria included those patients who had no evidence of infection on admission, but developed signs of infection after, at least, two days of hospitalization. Ethical approval to perform the study was obtained from the Egyptian Ministry of Health and Population. All the included patients consented to the collection of specimens from them before the study was initiated.

### Microbial identification

Gram-negative bacteria were identified using standard biochemical tests. We also used a Microscan Negative Identification panel Type 2 (NEG ID Type 2) (Dade Behring, West Sacramento, USA) to confirm the identification of gram-negative facultative bacilli. PID is an *in vitro *diagnostic product that uses fluorescence technology to detect bacterial growth or metabolic activity and thus can automatically identify gram-negative facultative bacilli to species level. The system is based on reactions obtained with 34 pre-dosed dried substrates which are incorporated into the test media in order to determine bacterial activity. The panel was reconstituted using a prompt inoculation system.

### Biochemical tests

In each Microscan NEG ID Type 2 kit, several biochemical tests were performed. These included carbohydrate fermentation tests, carbon utilization tests, and specific tests such as Voges Proskauer (VP), Nitrate reduction (NIT), Indole test, Esculine hydrolysis, Urease test, Hydrogen Sulphide production test, Tryptophan deaminase test, Oxidation-Fermentation test, and Oxidase test.

### Reagents

For the Microscan NEG ID Type 2 kit, reagents used were B1010-45A reagent (0.5% N, N-dimethyl-1-naphthylamine), B1015-44 reagent (Sulfanilic acid), B1010-48A reagent (10% ferric chloride), B1010-93 A reagent (40% Potassium hydroxide), B1010-42A reagent (5% α-naphthol), and B1010-41A reagent (Kovac's reagent).

### Antimicrobial susceptibility testing

Both automated and manual methods were used to detect antimicrobial susceptibility pattern of the isolates. The Microscan Negative Break Point combo panel type 12 (NBPC 12) automated system was used for antimicrobial susceptibility testing of gram-negative isolates. A prompt inoculation system was used to inoculate the panels. Incubation and reading of the panels were performed in the Microscan Walk away System. Kirby-Bauer technique (disc diffusion method) was also used to confirm resistant gram-negative isolates. Discs of several antimicrobial disks (Oxoid ltd., Basin Stoke, Hants, England) were placed on the surface of Muller Hinton agar plates followed by incubation at 35°C. Reading of the plates was carried out after 24 h using transmitted light by looking carefully for any growth within the zone of inhibition. Appropriate control strains were used to ensure the validity of the results. Susceptibility patterns were noted.

### Calculation of mortality rate

We only calculated attributable mortality which we defined as death within the hospital (or 28 days following discharge) [5,6], with signs or symptoms of acute infection (septic shock, multi-organ failure). Other deaths were considered deaths due to the underlying cancer and were excluded from calculations. In addition, patients with polymicrobial infections were excluded from the mortality rate calculation.

## Results

The main isolated gram-negative bacteria from all clinical specimens were *Klebsiella pneumonia *(31.2%; 241 out of 772 total gram-negative isolates) followed by *Escherichia coli *(22.2%). *Klebsiella pneumonia *was the main isolated gram-negative bacteria from sputum and throat (50.3% and 39.2% respectively) (Table 1). The main isolated gram-negative bacteria from blood were *Escherichia coli *(28.3%) and *Pseudomonas *species (16.7%). There was a significant proportion of cancer patients who developed SI. The most frequent gram-negative bacteria isolated from SI were *Klebsiella pneumonia *(25.4%), *Escherichia coli *(22.2%), and *Pseudomonas aeruginosa *(18.9%). The most commonly isolated gram-negative pathogens from urine and stool were *Escherichia coli *(37.8% and 36.4% respectively) and *Klebsiella pneumonia *(31.6% and 17.5% respectively) (Table 1).

**Table 1:** The microbial spectrum of gram-negative bacteria in different clinical specimens.

**Different species**	**Throat swab No(%)**	**Sputum No(%)**	**Chest tube No(%)**	**BAL No(%)**	**Pus No(%)**	**Urine No(%)**	**Stool No(%)**	**Blood No(%)**	**Total No(%)**
***Acinetobacter haemolyticus***	14(18.9)	12(6)	3(30)	-	9(4.9)	4(4.1)	1(0.7)	6(10)	49(6.4)

***Acinetobacter lwofii***	1(1.4)	3(1.5)	-	-	-	-	-	-	4(0.5)

***Acinetobacter *species (Total)**	15(20.3)	15(7.5)	3(30)	-	9(4.9)	4(4.1)	1(0.7)	6(10)	53(6.9)

***Citrobacter amaloniticus***	-	-	-	-	1(0.5)	-	-	-	1(0.1)

***Citrobacter freundi***	-	3(1.5)	-	-	6(3.2)	5(5.1)	6(4.2)	6(10)	26(3.4)

***Citrobacter *species (Total)**	-	3(1.5)	-	-	7(3.8)	5(5.1)	6(4.2)	6(10)	27(3.5)

***Enterobacter aerogenes***	2(2.7)	5(2.5)	1(10)	-	10(5.4)	2(2)	13(9.1)	2(3.3)	35(4.5)

***Enterobacter agglomerulance***	-	-	-	-	1(0.5)	-	2(1.4)	1(1.7)	4(0.5)

***Enterobacter cloacae***	6(8.1)	22(11)	-	-	5(2.7)	2(2)	7(4.9)	2(3.3)	44(5.7)

***Enterobacter gergovia***	-	-	-	-	1(0.5)	-	1(0.7)	-	2(0.3)

***Enterobacter *species (Total)**	8(10.8)	27(13.4)	1(10)	-	17(9.2)	4(4.1)	23(16.1)	5(8.3)	85(11)

***Escherichia coli***	7(9.5)	17(8.5)	-	-	41(22.2)	37(37.8)	52(36.4)	17(28.3)	171(22.2)

***Klebsiella ornithinolytica***	-	-	-	-	3(1.6)	2(2)	9(6.3)	1(1.7)	15(1.9)

***Klebsiella oxytoca***	-	1(0.5)	-	-	1(0.5)	-	3(2.1)	-	5(1.9)

***Klebsiella ozanae***	-	1(0.5)	-	-	2(1.1)	-	2(1.4)	-	5(1.9)

***Klebsiella pneumonia***	29(39.2)	101(50.3)	1(10)	-	47(25.4)	31(31.6)	25(17.5)	7(11.7)	241(31.2)

***Klebsiella rhinoscleroma***	-	3(1.5)	-	-	-	-	-	-	3(0.4)

***Klebsiella *species (Total)**	29(39.2)	106(52.7)	1(10)	-	53(28.7)	33(33.7)	39(27.3)	8(13.3)	269(34.8)

***Pseudomonas aeruginosa***	5(6.8)	10(5)	-	-	35(18.9)	7(7.1)	-	8(13.3)	65(8.4)

***Pseudomonas flourescence***	-	1(0.5)	-	-	3(1.6)	-	-	2(3.3)	6(0.8)

***Pseudomonas oryzihabitant***	-	-	-	-	-	-	3(2.1)	-	3(0.4)

***Pseudomonas stutzeri***	1(1.4)	3(1.5)	1(10)	-	-	-	-	-	5(0.6)

***Pseudomonas *species (Total)**	6(8.1)	14(7)	1(10)	-	38(20.5)	7(7.1)	3(2.1)	10(16.7)	79(10.2)

***Serratia fonticola***	1(1.4)	2(1)	-	-	2(1.1)	1(1)	4(2.8)	-	10(1.3)

***Serratia liquificans***	2(2.7)	1(0.5)	-	-	-	-	-	-	3(0.4)

***Serratia marcescens***	-	-	-	-	2(1.1)	-	-	-	2(0.3)

***Serratia odorifera***	-	-	-	-	1(0.5)	2(2)	2(1.4)	-	5(0.7)

***Serratia plymuthica***	1(1.4)	-	-	-	-	-	-	-	1(0.1)

***Serratia rubidae***	2(2.7)	2(1)	-	-	-	-	-	-	4(0.5)

***Serratia *species (Total)**	6(8.1)	5(2.5)	-	-	5(2.7)	3(3.1)	6(4.2)	-	25(3.2)

**Other gram-negative species**	3(4.1)	14(7)	4(40)	1(100)	15(8.1)	5(5.1)	13(9.1)	8(13.3)	63(8.2)

**Total gram-negative species**	74(9.6)	201(26)	10(1.3)	1(0.1)	185(24)	98(12.7)	143(18.5)	60(7.8)	772(100)

A number of less-frequent gram negative bacteria were isolated and identified (*Chromobacterium violacum*, *Burkholderia cepacia, Kluyvera ascorbata, Stenotrophomonas maltophilia, Yersinia pseudotuberculosis*, and *Salmonella arizona*). In addition, there was a low frequency of enteric infections as evidenced by the low prevalence of *Salmonella*, *Shigella*, and *Yersinia *species (Table 2).

**Table 2:** The microbial spectrum of less frequent gram-negative bacteria in different clinical specimens.

**Different species**	**Throat swab**	**Sputum**	**Chest tube**	**BAL**	**Pus**	**Urine**	**Stool**	**Blood**	**Total No(%)**
***Aeromonas hydrophila***	-	-	-	-	1	-	-	-	1(1.6)

***Alcaligenes xylosoxidans***	-	-	-	-	1	1	-	-	2(3.2)

***Bordetella bronchiseptica***	-	1	-	-	-	-	-	-	1(1.6)

***Burkholderia cepacia***	1	2	-	1	2	-	-	-	6(9.5)

***CDC gp IV C-2***	-	-	-	-	1	-	-	1	2(3.2)

***Cedecea lapagei***	-	-	-	-		-	-	1	1(1.6)

***Chryseobacterium indologenes***		1	-	-	-	-	-	-	1(1.6)

***Chryseobacterium meningosepticum***	-	-	1	-	1	-	1	-	3(4.8)

***Chromobacterium violacum***	1	1	-	-	4	1	-	-	7(11.1)

***Hafnia alvei***	-	-	-	-	1	-	1	-	2(3.2)

***Kluyvera ascorbata***	-	2	-	-	-	-	3	-	5(7.9)

***Morganella morgani***	-	2	-	-	-	1	-	-	3(4.8)

***Proteus mirabilis***	-	-	-	-	1	-	-	-	1(1.6)

***Proteus penneri***	-	-	-	-	-	-	-	2	2(3.2)

***Proteus vulgaris***	-	-	-	-	1	-	-	-	1(1.6)

***Providencia rettgeri***	-	-	-	-	-	1	-	-	1(1.6)

***Providencia stuarti***	-	-	-	-	1	-	-	-	1(1.6)

***Salmonella arizona***	-	-	-	-	-	-	2	1	3(4.8)

***Salmonella choleraesuis***	-	-	-	-	-	-	1	-	1(1.6)

***Salmonella Paratyphi A***	-	-	-	-	-	-	1	-	1(1.6)

***Shigella *species**	-	-	-	-	-	-	4	-	4(6.4)

***Stenotrophomonas maltophilia***	1	3	1	-	-	-	-	-	5(7.9)

***Vibrio alginolyticus***	-	1	-	-	-	-	-	-	1(1.6)

***Vibrio fluvialis***	-	-	1	-	-	-	-	-	1(1.6)

***Yersinia enterocolitica***	-	1	-	-	-	-	-	1	2(3.2)

***Yersinia pseudotuberculosis***	-	-	1	-	-	-	-	2	3(4.8)

***Yersinia ruckeri***	-	-	-	-	-	1	-	-	1(1.6)

***Yokenella regensburgei***	-	-	-	-	1	-	-	-	1(1.6)

**Total No(%)**	3(4.8)	14(22.2)	4(6.4)	1(1.6)	15(23.8)	5(7.9)	13(20.6)	8(12.7)	63(100)

Out of 772 total gram-negative isolates, 286 isolates (37.1%) were isolated from Respiratory Tract Infections (RTI). Out of 286 gram-negative isolates from RTI, 242 isolates were obtained from leukemic patients (84.6%), whereas only 44 isolates were obtained from solid-tumor patients (15.4%). Out of 143 gram-negative isolates from GITI, 123 isolates were obtained from leukemic patients (86%), whereas only 20 isolates were obtained from solid-tumor patients (14%). Out of 60 gram-negative isolates from BSI, 43 isolates were obtained from leukemic patients (71.67%), whereas only 17 isolates were obtained from solid-tumor patients (28.33%). Out of 98 gram-negative isolates from UTI, 77 isolates were isolated from leukemic patients (78.6%), whereas only 21 isolates were obtained from solid-tumor patients (21.4%). All the 185 gram-negative isolates from SI were isolated from solid-tumor patients (Table 3).

**Table 3:** The spectrum of gram-negative pathogens in various infection sites in leukemic and solid-tumor patients.

**Gram negative isolates**	**RTI**	**GITI**	**BSI**	**UTI**	**SI**	**Total**
**Leukemic patients**	242	123	43	77	-	485

**Solid-tumor patients**	44	20	17	21	185	287

**Total**	286	143	60	98	185	772

RTI = Respiratory Tract Infections, GITI = Gastro-Intestinal Tract Infections, SI = Skin Infections, BSI = Blood Stream Infections, UTI = Urinary Tract Infections

Results in table 4 indicated that in both leukemic patients and solid-tumor cancer patients, gram-negative bacteria causing nosocomial UTI were mainly *Escherichia coli *(39% in case of leukemic patients, 33.3% in case of solid-tumor cancer patients) and *Klebsiella pneumoniae *(27.3% in case of leukemic patients, 47.6% in case of solid-tumor cancer patients). In both leukemic patients and solid-tumor cancer patients, gram-negative bacteria causing nosocomial RTI were mainly *Klebsiella pneumoniae *(48.4% in case of leukemic patients, 27.3% in case of solid-tumor cancer patients). *Escherichia coli *was the main gram-negative pathogen causing BSI in solid-tumor patients (70.6%) and GITI in leukemic patients (34.2%). Several organisms contributed to BSI in leukemic patients (such as, *Klebsiella pneumonia, Pseudomonas aeruginosa, Citrobacter freundi, Acinetobacter baumannii/haemolyticus*, and *Escherichia coli)*. In patients with solid-tumor malignancies, the most frequent nosocomical infections caused by gram-negative bacteria were SI (185 isolates; 64.5% of gram-negative nosocomial infections in solid-tumor patients) (Table 3). *Klebsiella pneumonia *(25.4%), *Escherichia coli *(22.2%), and *Pseudomonas aeruginosa *(18.9%) were the most predominant gram-negative bacteria in SI in solid-tumor cancer patients (Table 4). It is noteworthy that no gram negative isolates were recovered from SI in leukemic patients (Table 3).

**Table 4:** The spectrum of predominant gram-negative bacteria in Bloodstream Infections (BSI), Urinary Tract Infections (UTI), Respiratory Tract Infections (RTI), Gastro-Intestinal Tract Infections (GITI), and Skin Infections (SI) of leukemic and solid-tumor patients.

	**Patients with Leukemia No(%)**				**Solid-tumor Patients No(%)**				
**Species**	**BSI**	**UTI**	**RTI**	**GITI**	**BSI**	**UTI**	**SI**	**RTI**	**GITI**

***Acinetobacter baumannii/haemolyticus***	6(14)	4(5.2)	26(10.7)	1(0.8)	-	-	9(4.9)	3(6.8)	-

***Acinetobacter lwoffii***	-	-	4(1.7)	-	-	-			-

***Aeromonas hydrophila***	-	-	-	-	-	-	1(0.5)	-	-

***Alcaligenes xylosoxidans***	-	1(1.3)	-	-	-	-	1(0.5)	-	-

***Bordetella bronchiseptica***	-	-	1(0.4)	-	-	-	-	-	-

***Burkholderia cepacia***	-	-	3(1.2)	-	-	-	2(1.1)	1(2.3)	-

**CDC gp IV C-2**	1(2.3)	-	-	-	-	-	1(0.5)	-	-

***Cedecea lapagei***	1(2.3)	-	-	-	-	-	-	-	-

***Chromobacterium violaceum***	-	1(1.3)	2(0.8)	-	-	-	4(2.2)	-	-

***Chryseobacterium indologenes***	-	-	1(0.4)	-	-				-

***Chryseobacterium meningosepticum***	-	-	-	1(0.8)	-	-	1(0.5)	1(2.3)	-

***Citrobacter amaloniticus***	-	-	-	-	-	-	1(0.5)	-	-

***Citrobacter freundi***	6(14)	4(5.2)	3(1.2)	6(4.9)	-	1(4.8)	6(3.2)	-	1(5)

***Enterobacter aerogenes***	2(4.7)	-	7(2.9)	13(10.6)	-	2(9.5)	10(5.4)	1(2.3)	1(5)

***Enterobacter agglomerans***	1(2.3)	-	-	2(1.6)	-	-	1(0.5)	-	-

***Enterobacter cloacae***	2(4.7)	2(2.6)	26(10.7)	7(5.7)	-	-	5(2.7)	3(6.8)	2(10)

***Enterobacter gergoviae***	-	-	-	1(0.8)	-	-	1(0.5)	-	-

***Escherichia coli***	5(11.6)	30(39)	13(5.4)	42(34.2)	12(70.6)	7(33.3)	41(22.2)	9(20.5)	7(35)

***Hafnia alvei***	-	-	-	1(0.8)	-	-	1(0.5)	-	-

***Klebsiella ornithinolytica***	1(2.3)	2(2.6)	-	5(4.1)	-	-	3(1.6)	-	2(10)

***Klebsiella oxytoca***	-	-	1(0.4)	3(2.4)	-	-	1(0.5)	-	1(5)

***Klebsiella ozanae***	-	-	-	2(1.6)	-	-	2(1.1)	1(2.3)	1(5)

***Klebsiella pneumoniae***	6(14)	21(27.3)	118(48.8)	19(15.4)	1(5.9)	10(47.6)	47(25.4)	12(27.3)	4(20)

***Klebsiella rhinoscleroma***	-	-	1(0.4)	-	-	-	-	2(4.6)	-

***Kluyvera ascorbata***	-	-	2(0.8)	3(2.4)	-	-	-	-	-

***Morganella morgani***	-	1(1.3)	2(0.8)	-	-	-	-	-	-

***Proteus mirabilis***	-	-	-	-	-	-	1(0.5)	-	-

***Proteus penneri***	2(4.7)	-	-	-	-	-	-	-	-

***Proteus vulgaris***	-	-	-	-	-	-	1(0.5)	-	-

***Providencia rettgeri***	-	-	-	-	-	-	1(0.5)	-	-

***Providencia stuarti***	-	1(1.3)	-	-	-	-	-	-	-

***Pseudomonas aeruginosa***	6(14)	6(7.8)	11(4.6)	-	2(11.8)	1(4.8)	35(18.9)	6(13.6)	-

***Pseudomonas fluorescens***	-	-	1(0.4)	-	2(11.8)	-	3(1.6)	-	-

***Pseudomonas oryzihabitans***	-	-	-	3(2.4)	-	-	-	-	-

***Pseudomonas stutzeri***	-	-	4(1.7)	-	-	-	-	1(2.3)	-

***Salmonella *species**	1(2.3)	-	-	4(3.3)	-	-	-	-	-

***Serratia fonticola***	-	1(1.3)	3(1.2)	4(3.3)	-	-	2(1.1)	-	1(5)

***Serratia liquefaciens***	-	-	2(0.8)	-	-	-	-	1(2.3)	-

***Serratia marcescens***	-	-	-	-	-	-	2(1.1)	-	-

***Serratia odorifera***	-	2(2.6)	-	2(1.6)	-	-	1(0.5)	-	-

***Serratia plymuthica***	-	-	1(0.4)	-	-	-	-	-	-

***Serratia rubidaea***	-	-	4(1.7)	-	-	-	-	-	-

***Shigella *species**	-	-	-	4(3.3)	-	-	-	-	-

***Stenotrophomonas maltophilia***	-	-	4(1.7)	-	-	-	-	1(2.3)	-

***Vibrio alginolyticus***	-	-	1(0.4)	-	-	-	-	1(2.3)	-

***Yersinia enterocolitica***	1(2.3)	-	1(0.4)	-	-	-	-	-	-

***Yersinia Pseudotuberculosis***	2(4.7)	-	-	-	-	-	-	1(2.3)	-

***Yersinia ruckeri***	-	1(1.3)	-	-	-	-	-	-	-

***Yokenella regensburgei***	-	-	-	-	-	-	1(0.5)	-	-

**Total**	43(100)	77(100)	242(100)	123(100)	17(100)	21(100)	185(100)	44(100)	20(100)

The antimicrobial resistance patterns of different gram-negative isolates from cancer patients were examined. Isolates of *Escherichia coli, Klebsiella, Enterobacter*, *Pseudomona, and Acinetobacter *species were resistant to most antibiotics tested including non-β-lactam antibiotics such as aminoglycosides (gentamicin) and quinolones (ciprofloxacin, levofloxacin). In addition, isolates exhibited simultaneous resistance to more than one non β-lactam drug (Tables 5 and 6).

**Table 5:** Antimicrobial susceptibility *of Escherichia coli*, *Klebsiella, and Enterobacter *species

	***Escherichia coli***				***Klebsiella *species**				***Enterobacter species***			
**Antibiotic**	**B**	**S**	**I**	**R**	**B**	**S**	**I**	**R**	**B**	**S**	**I**	**R**

**Amikacin**	32	81.5	5.6	13	32	62.8	5.8	31.4	32	45.5	6.1	48.5

Amx-Clav*	16/8	38.7	30.3	31	16/8	46.9	18.6	34.5	16/8	3	12.1	84.5

**Ampicillin**	16	15.9	7.1	77	16	1.8	0	98.2	16	3.3	0	96.7

Amp-Sul**	16/8	6.9	0	93.2	16/8	25.5	3.1	71.4	16/8	0	0	100

**Aztereonam**	16	38.7	5.4	55.9	16	40.6	2.9	56.5	16	16.7	0	83.3

**Cefazolin**	16	21.9	2.1	76	16	25.2	2.8	71.9	16	0	0	100

**Cefepime**	16	38.6	1.2	60.2	16	35.6	5.1	59.3	16	26.3	5.3	68.4

**Cefopyrazon**	32	32.2	1.2	66.7	32	37.4	3.6	59	32	11.8	5.9	82.4

**Cefotaxime**	16	32.3	1.5	66.2	32	37.3	3	59.6	32	16	16	68.4

**Cefotetan**	32	82.1	5.8	12.2	32	86.5	3.1	16.4	32	35.3	14.7	50

**Cefoxitin**	16	61.6	11.6	26.7	16	57.4	14.7	27.9	16	11.1	0	88.9

**Ceftazidime**	16	40.5	3.8	55.7	16	52	0	48	16	14.3	7.1	78.6

**Ceftizoxime**	32	37.8	8.5	53.6	32	42.4	4.6	53	32	6.3	12.5	81.3

**Ceftriaxone**	16	29.6	1.3	69.1	16	35.3	4.2	60.5	32	12.5	12.5	75

**Cefuroxime**	16	24.4	4.5	71.2	16	32.7	4.4	62.8	16	7.7	7.7	84.6

**Cephalothin**	16	7.1	3.4	90.5	16	25	4.4	70.6	16	0	0	100

**Ciprofloxacin**	2	33.7	0.6	55.9	2	60	4	36	2	69.7	0	30.3

**Gatifloxacin**	4	33.9	1.8	64.3	4	60.5	7	32.6	4	58.4	8.3	33.3

**Gentamicin**	8	42.3	1.8	66.7	8	50.4	0.8	48.8	4	38.7	6.5	54.8

**Imipenem**	8	91.2	0.7	8	8	85.1	1	13.9	8	66.7	11.1	22.2

**Levofloxacin**	4	34.4	2.7	62.9	4	63.2	6.1	30.7	4	80	3.3	16.7

**Meropenem**	8	50.5	0	49.5	8	80.5	0	30.7	8	75	7.1	17.9

**Mezlocillin**	64	3	3	94	64	0	2.9	97.1	64	1	2	97

**Netilmicin**	16	53.6	18.8	27.5	16	51.6	1.6	46.8	16	58.8	11.8	29.4

**Piperacillin**	64	3.4	2.3	94.3	64	2.7	2.7	94.6	64	11.8	5.9	82.4

Pip-Taz***	64	45.3	15.6	39.1	32	45.7	11.4	42.9	64	29.4	5.9	64.7

Sul-Tri****	16	19.9	0	80.1	16	34.7	0	65.3	16	23.5	0	76.5

**Tetracycline**	8	14.3	1.1	84.6	8	44.8	4.5	50.8	8	23.5	11.8	64.7

**Ticarcillin**	64	6.3	2.5	91.1	64	4.2	1.4	94.4	64	0	12.5	87.5

Tic-Cla*****	64	27.9	27.9	44.1	64	44.3	11.3	44.3	64	28	12	60

**Tobramycin**	8	35.1	5.8	59.1	8	42.2	5.2	52.6	8	39.3	7.1	53.6

B = Breakpoint S = Susceptible I = Intermediate R = Resistant

* Amoxicillin-Clavulanate ** Ampicillin-Sulbactam *** Piperacillin-Tazobactam ****Sulfamethoxazole- Trimethoprim *****Ticarcillin/Clavulanate

**Table 6:** Antimicrobial susceptibility of *Pseudomonas and Acinetobacter *species

	***Pseudomonas *species**				***Acinetobacter *species**			
**Antibiotic**	**B**	**S**	**I**	**R**	**B**	**S**	**I**	**R**

Amikacin	32	44.2	3.9	51.9	32	44.9	6.1	49

Amp-Sul*	16/8	37	10	53	16/8	35.9	12.8	51.3

Aztereonam	16	10.5	7.9	81.6	16	10	12.5	77.5

Cefepime	16	38.9	5.6	55.6	16	25	12.5	62.5

Cefopyrazon	32	13.2	0	86.8	32	11.4	0	88.6

Cefotaxime	16	4.3	10.6	85.1	32	11.1	15.6	73.3

Cefotetan	32	25	12.5	62.5	32	36.5	4.5	59

Ceftazidime	16	28	2	70	16	29	5	66

Ceftizoxime	32	2.9	11.4	85.7	32	17.7	5.9	76.5

Ceftriaxone	16	4.1	16.3	79.6	32	23.9	15.2	60.9

Ciprofloxacin	2	42.3	3.9	53.9	2	52.1	4.2	43.8

Gentamicin	8	35.9	11.3	52.8	4	42.6	4.3	53.2

Imipenem	8	54	6	40	8	54.6	4.6	40.9

Levofloxacin	4	51.9	1.9	46.2	4	58.7	2.2	39.1

Meropenem	8	50.9	11.3	37.7	8	55	5	40

Mezlocillin	64	6.9	0	93	64	7	0	93

Netilmicin	16	30.6	13.9	55.6	16	53.1	6.3	40.6

Piperacillin	64	10.5	2.6	86.8	64	15.4	15.4	69.2

Pip-Taz**	32	40	6.7	53.3	64	47.7	6.8	45.5

Sul-Tri***	16	40	0	60	16	41.3	0	58.7

Tetracycline	8	21.1	10.5	68.4	8	36.4	6.1	57.6

Ticarcillin	64	8.3	0	91.7	64	21.2	12.1	66.7

Tic-Cla****	64	24.5	4.1	71.4	64	17.1	14.6	68.3

Tobramycin	8	52.8	1.9	45.3	8	54.4	2.2	43.5

B = Breakpoint S = Susceptible I = Intermediate R = Resistant

*Ampicillin-Sulbactam **Piperacillin-Tazobactam *** Sulfamethoxazole- Trimethoprim **** Ticarcillin/Clavulanate

*Escherichia coli *exhibited slightly higher resistance to levofloxacin (62.9%) and gatifloxacin (64.3%) than to ciprofloxacin (55.9%). By contrast, *Klebsiella pneumonia *exhibited slightly lower resistance to levofloxacin (30.7%) and gatifloxacin (32.6%) than to ciprofloxacin (36%). A similar trend was seen with *Pseudomonas *and *Acinetobacter *species which both exhibited lower resistance to levofloxacin than to ciprofloxacin. For *Enterobacter *species, resistance to levofloxacin (16.7%) was significantly lower than to gatifloxacin (33.3%) or ciprofloxacin (30.3%) (Tables 5 and 6).

Carbapenems are highly potent broad-spectrum β-lactams to which resistance of gram-negative bacteria had been previously reported [7]. Resistance to imipenem was observed with *Acinetobacter *species (40.9%), *Pseudomonas *(40%), *Enterobacter *(22.2%), *Klebsiell*a (13.9%), and *Escherichia coli *(8%) (Tables 5 and 6). Aztereonam is a monobactam antibiotic with antimicrobial activity against gram-negative bacilli such as *Pseudomonas aeruginosa *[8]. Isolates of *Escherichia coli, Klebsiella *species, *Enterobacter *species, *Pseudomonas *species, and *Acinetobacter *species exhibited resistance to aztereonam at the following respective percentages of resistance: 55.9%, 56.5%, 83.3%, 81.6%, and 77.5% (Tables 5 and 6).

Gram-negative isolates were highly resistant to cefotaxime and ceftazidime. *Escherichia coli *exhibited 66.2% and 55.7% resistance to Cefotaxime and Ceftazidime. The percentage resistance to cefotaxime and ceftazidime was also high in *Klebsiella*, *Enterobacter*, *Pseudomonas*, and *Acitenobacter *isolates (Tables 5 and 6). In addition, 70.2% of *Pseudomonas *species isolates exhibited simultaneous resistance to cefotaxime and ceftazidime. Other gram-negative species also exhibited similar high rates of resistance to both cefotaxime and ceftazidime (Table 7).

**Table 7:** Percentage of potential Extended-spectrum β-lactamase (ESβL)-producing gram-negative bacteria and percentage mortality attributed to each of the indicated species of gram-negative bacteria

**Species**	**Resistance to both Cefotaxime and Ceftazidime (Potential ESβL-producers)**	**In-hospital Mortality Rate**
***Acinetobacter***	62.2%	16%

***Escherichia coli***	54%	11.9%

***Enterobacter *species**	64%	15%

***Klebsiella *species**	44.8%	8.7%

***Pseudomonas *species**	70.2%	34.1%

***Serratia *species**	62.5%	12.5%

It should be noted that the use of Tazobactam (β-lactamase inhibitor) enhanced the activity of piperacillin against *Acinetobacter*, *Pseudomonas, Enterobacter, Klebsiell*a, and *Escherichia coli*. Similarly, the use of Clavulanate restored the activity of Ticarcillin against *Pseudomonas, Enterobacter, Klebsiell*a, and *Escherichia coli *(Tables 5 and 6).

*Escherichia coli *isolates were highly susceptible to imipenem (8% resistance), cefotetan (12.2% resistance), and amikacin (13% resistance). *Klebsiella *species isolates were susceptible to imipenem (13.9% resistance), and cefotetan (16.4% resistance). *Enterobacter *species isolates were susceptible to levofloxacin (16.7% resistance) and meropenem (17.9% resistance). *Pseudomonas *species isolates were resistant to most antibiotics tested, with meropenem being the most active antibiotic against *Pseudomonas *(37.7% resistance). *Acinetobacter *species isolates were resistant to most antibiotics tested, with levofloxacin being the most active antibiotic against *Pseudomonas *(39.1% resistance) (Tables 5 and 6).

Results in Table 7 demonstrated the mortality rate was higher among patients with nosocomial *Pseudomonas *infections (34.1%) than other bacterial infections. It is noteworthy that *Pseudomonas *isolates exhibited significant resistance to both cefotaxime and ceftazidime (70% resistance). By contrast, *Klebsiella *species, which were 44.8% resistant to both cefotaxime and ceftazidime, caused only 8.7% mortality.

## Discussion

The goal of this study was to characterize the microbial spectrum and antibiotic susceptibility profile of gram-negative bacteria in cancer patients. The most frequently isolated gram-negative bacteria from all clinical specimens were *Klebsiella pneumonia *followed by *Escherichia coli *(Table 1). Other studies reported that *Escherichia coli *and *Klebsiella *species were the most frequently isolated gram-negative pathogens in nosocomial infections from cancer and non-cancer patients [9,10]. Similarly, Bilal *et al *reported that *Klebsiella pneumonia *was the most common isolate in their hospital in Saudia Arabia [11].

*Klebsiella pneumonia *was the main isolated gram-negative bacteria from sputum and throat (Table 1). This is consistent with the work of Hoheisel *et al *in Germany who reported that *Klebsiella *species were among the most frequent gram-negative isolates from RTI [12]. Results in table 1 indicated that the main isolated gram-negative bacteria from blood were *Escherichia coli *and *Pseudomonas *species (Table 1). Other studies also reported *Escherichia coli *and *Pseudomonas *species to be among the most prevalent organisms causing bloodstream infections in USA [13].

In the present study, 18% of cancer patients developed SI (data not shown). This is consistent with other studies which reported significant surgical site infection rates in cancer treatment centers [14,15]. As shown in table 1, the most commonly isolated gram-negative bacteria from SI were *Klebsiella pneumonia, Escherichia coli*, and *Pseudomonas aeruginosa*. Vilar-Compte *et al *reported that *Escherichia coli *and *Pseudomonas *species were the most commonly isolated bacteria from surgical site infections at a cancer center in Mexico [15]. The main isolated organisms from urine were *Escherichia coli *and *Klebsiella pneumonia *(Table 1). This is reminiscent of the study by Espersen *et al *who demonstrated that UTI due to *Escherichia coli *were the most frequent infections in patients with myelomatosis [16].

In addition to the present study, the isolation of *Burkholderia cepacia *and other less-frequent gram-negative bacteria had been reported in other studies of nosocomial infections in cancer and non-cancer patients [17-19] (Table 2). The low prevalence of *Salmonella*, *Shigella*, and *Yersinia *species reported in our study was not unusual in the realm of nosocomial infections in cancer patients. In his study on patients with acute leukemia, Gorschluter *et al *reported low frequency of enteric infections by *Salmonella*, *Shigella*, *Yersinia*, and *Campylobacter *[20].

As in tables 5 and 6, all gram-negative species examined were highly resistant to third-generation cephalosporins. Reports from Korea and other parts of the world indicted that nosocomial infections caused by *Enterobacter*, *Citrobacter*, and *Serratia *species were also resistant to third generation cephalosporins [21].

Isolates producing ESβL confer resistance to all β-lactam agents and to other classes of antimicrobial agents, such as amino glycosides and flouroquinolones, thus making it difficult to treat infections they produce [22]. Reports indicate a significant increase in ESβL-producers in recent years [23]. Invasive procedures, specifically catheterization, prolonged hospital stay and confinement in an oncology unit were found to be associated with ESβL production [24]. Ceftazidime and cefotaxime resistance are potential markers for the presence of Extended-Spectrum β lactamases (ESβL). Aztreonam resistance is also a potential marker for the presence of an ESβL-producing organism. Levels of resistance to aztereonam among gram-negative isolates (Tables 5 and 6) were higher than those reported few years ago in Egypt [25]. In addition, there were high percentages of cefotaxime/ceftazidime-resistant gram-negative isolates. All of this suggested ESβL production (Tables 5, 6, 7). However, further confirmatory tests are needed to confirm the presence of ESβL enzymes in such isolates. This is an important future avenue specially that previous reports suggested that ESβL-producing strains were endemic in Egypt [25].

Compared with second-generation quinolones (ciprofloxacin), the newest fluoroquinolones (levofloxacin, gatifloxacin) have enhanced activity against gram-positive bacteria with only a minimal decrease in activity against gram-negative bacteria [26]. However, the newer generation quinolones are still quite active against most Enterobacteriaceae (such as *Enterobacter, Escherichia, Klebsiella*) and non-fermentative gram-negative bacilli (such as *Acinetobacter*) with the exception of *Pseudomonas aeruginosa *[27]. Results in tables 5 and 6 demonstrated that whereas *Klebsiella*, *Pseudomonas*, and *Acinetobacter *were relatively more susceptible to newer quinolones than ciprofloxacin, *Escherichia coli *was more susceptible to ciprofloxacin. *Enterobacter *was particularly susceptible to levofloxacin. Thus, an older or newer quinolone may be more active depending on the particular gram-negative species involved.

Previous studies in Egypt reported that resistance to imipenem was totally absent or very low [25,28]. A similar observation was made in a study in Turkey [29]. Other studies in Turkey, Italy, and France reported the presence of low levels of resistance to imipenem [30-33]. *Acinetobacter *and *Pseudomonas *species exhibited the highest resistance levels to imipenem. *Enterobacter *still exhibited considerable resistance to imipenem. *Escherichia coli *and *Klebsiell*a exhibited lower, but still noticeable, resistance to imipenem. To our knowledge, this is the first study which reports significant levels of imipenem resistance in Egypt.

*Escherichia coli *isolates were highly resistant to ampicillin, ampicillin-sulbactam, aminoglycosides, and other antibiotics. El Kholy *et al *reported that *Escherichia coli *isolates from cancer patients in Egypt exhibited a low susceptibility pattern [25].

In a study conducted in Turkey, *Acinetobacter baumannii *was resistant to most antibiotics tested except meropenem, tobramycin, and imipenem [34]. Results in Table 6 showed that *Acinetobacter *species, as well as *Pseudomonas *species, were highly resistant to ceftazidime, aztereonam, piperacillin, and amino glycosides as was reported in other studies [35,36]. Some investigators noticed that geographic differences affected the resistance patterns of gram-negative bacteria such as *Acinetobacter *species [36]. In such a case, local surveillance will be important in order to determine the most adequate therapy for infections caused by such organisms.

Nosocomial outbreaks of the gram-negative pathogen *Enterobacter cloacae *were previously reported [37,38]. Our study confirmed previous reports which indicated that *Enterobacter *species isolated from hospitalized cancer patients from Egypt were highly resistant to ceftazidime, cefotaxime and aztereonam [25].

The phenomenon of multi drug resistant pathogens had emerged in Egypt and worldwide in recent years due to excessive antibiotic misuse [25,39]. Thus, Pathogens resistant to cephalosporins (third or fourth generation), carbapenems, aminoglycosides, and fluoroquinolone had emerged [39]. This study showed that gram-negative isolates can be resistant to more than one non β-lactam drug.

As indicated in table 7, the mortality rate associated with *Pseudomonas *infections in cancer patients was 34.1%. Previous reports also indicated high mortality rates (22%–33%) associated with *Pseudomonas *and *Escherichia coli *infections in immuno-compromised patients [40,41]. Similarly, the mortality rate (16%) attributed to *Acinetobacter *species infections was not very different from mortality rates attributed to *Acinetobacter *species infections in other reports (14–20%) [42,43].

The high levels of antimicrobial resistance in gram-negative bacteria can be attributed to antibiotic misuse in Egypt. Policies on the control of antibiotic usage have to be enforced and implemented to avoid the evolution of newer generations of pathogens with higher resistance, not only to the older generation drugs, but also to the relatively new ones. In addition, the entire microbial spectrum in various infection sites, and not just bloodstream pathogens, should be taken into account when initiating empirical antibiotic therapy.

## Abbreviations

RTI: Respiratory Tract Infections; SI: Skin Infections; UTI: Urinary Tract Infections; GITI: Gastro-intestinal Tract Infections; BSI: Bloodstream Infections

## Competing interests

The authors declare that they have no competing interests.

## Authors' contributions

HMA and AE contributed to conception and design, provision of study materials or patients, collection and assembly of data, data analysis and interpretation and manuscript writing. All authors read and approved the final manuscript.
